# Mechanisms of Epstein-Barr virus-associated autoimmunity: a comparative overview

**DOI:** 10.3389/fimmu.2026.1891373

**Published:** 2026-07-21

**Authors:** Fathima Shabnam, Gulfaraz Khan

**Affiliations:** 1 Department of Medical Microbiology and Immunology, College of Medicine and Health Sciences, United Arab Emirates University, Al Ain, United Arab Emirates; 2 Zayed Bin Sultan Center for Health Sciences, United Arab Emirates University, Al Ain, United Arab Emirates

**Keywords:** EBV, immune system, molecular mimicry, MS, RA, SLE

## Abstract

Epstein–Barr virus (EBV) is a ubiquitous herpesvirus, increasingly implicated in the pathogenesis of several autoimmune diseases, such as multiple sclerosis (MS), systemic lupus erythematosus (SLE), and rheumatoid arthritis (RA). These diseases have both, shared and disease-specific immunopathogenic pathways involving EBV. Shared mechanisms include the role of EBV latent proteins in triggering immune dysfunction, molecular mimicry between viral and self-antigens, infection of autoreactive B-cells, and dysfunction of type 1 interferon (IFN-1) responses. In MS, EBV is associated with a CNS-compartmentalized CD8^+^ T-cell responses, molecular mimicry with neural antigens, and formation of meningeal tertiary lymphoid structures. In SLE, EBV contributes to systemic autoimmunity through mimicry with multiple autoantigens, recurrent viral reactivation, and IFN-driven multi-organ inflammation. In RA, EBV promotes the formation of synovial ectopic lymphoid structures, enhances anti-citrullinated protein antibody production, and drives proinflammatory cytokine dysregulation. Host genetic variations, particularly in HLA alleles, further modulate susceptibility by influencing antigen presentation, viral control and autoreactive T-cell responses. Rather than acting as a uniform and consistent trigger, EBV appears to function as a context-dependent immunological modifier whose pathogenic effects are influenced by factors such as the timing of infection, tissue microenvironment, HLA-associated genetic background, and other environmental exposures. Unravelling the details of these mechanisms may inform targeted preventive and therapeutic strategies for EBV-associated autoimmune diseases.

## 1 Introduction

Epstein-Barr Virus (EBV), also known as human herpesvirus 4 (HHV-4), is arguably one of the most ubiquitous human viruses, infecting at least 90% of adults worldwide (1). It was first isolated from a case of Burkitt’s lymphoma in 1964, indicating that this virus could be oncogenic, a notion which was subsequently shown to be correct (2). A few years later, another dimension to EBV emerged when researchers unexpectedly found an association between the virus and autoimmune diseases when studying lymphomas (3). This multifaceted behavior of EBV has ever since remained an area of scientific interest.

Structurally, EBV, like other herpesviruses, is composed of a lipid envelope, tegument, and nucleocapsid (4). The outer lipid bilayer contains viral glycoproteins that regulate cellular tropism and membrane fusion. Beneath the envelope lies a pleomorphic tegument layer including the capsid-associated tegument complex, which links the envelope to the nucleocapsid (4–6). The icosahedral nucleocapsid encloses a linear double-stranded DNA genome and several dozen microRNAs (7, 8). Following infection, annealing of terminal repeat sequences enables ligation of genomic ends, resulting in circularization of the viral genome and establishment of a nuclear episomal form.

The life cycle of EBV is governed by dynamic interactions between the virus and the host immune system to initiate primary infection, establish latency, and periodic reactivation resulting in the production of new virions. Primary infection is usually asymptomatic in infancy and early childhood, whereas delayed exposure in adolescents and young adults via salivary exchange can result in infectious mononucleosis (“kissing disease”) (9). Other transmission routes include sexual contact, organ transplantation, and blood transfusion. Following infection, EBV establishes latency in circulating peripheral blood B-lymphocytes with restricted viral gene expression and minimal virion production, enabling the virus to evade the immune system and establish life-long persistence (10).

**Figure 1:**
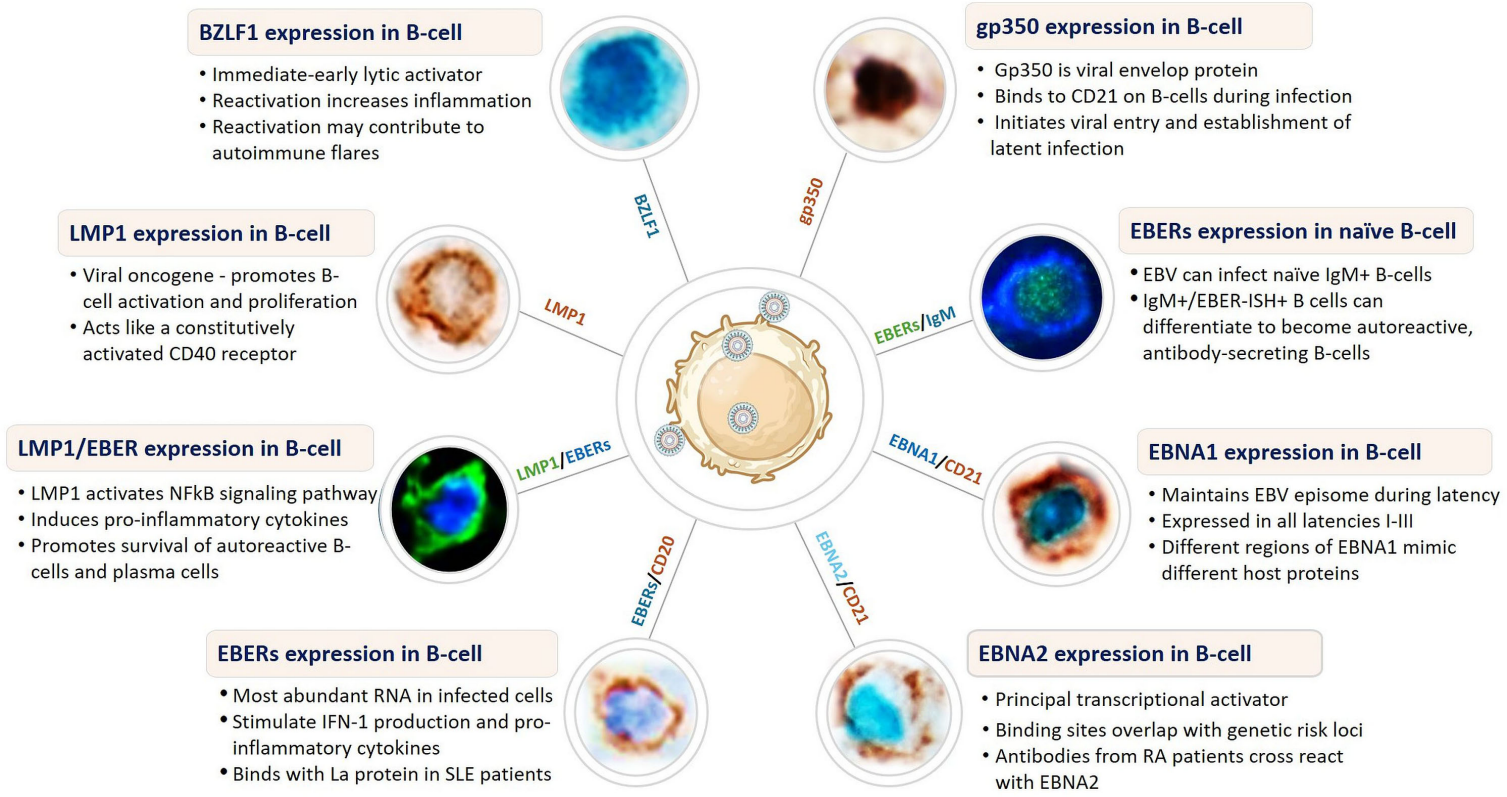
EBV gene expression in B-cells and potential role in autoimmunity. EBV infects B-cells via the binding of the envelop glycoprotein gp350 with cellular CD21/C3d receptor. The virus can infect naïve IgM positive B-cells. In these cells the virus can expression a range of different latent proteins as well as non-coding RNAs (EBERs) and miRNAs. In immunocompetent hosts, naïve cells can differentiate into memory B-cells in lymphoid tissues and the virus establishes life-long latency. On reactivation, the virus expresses a limited set of latent proteins including EBNA1, EBNA2 and LMP1. Some of these proteins have been implicated in the pathogenesis of autoimmune diseases like MS, SLE and RA. All the immunohistochemistry (IHC) and EBER *in situ* hybridization (EBER-ISH) staining were performed on rabbit spleen tissues (17). Individual stained cells were cropped from the larger images and used to produce this figure.

In infected B-cells, EBV can express up to six Epstein-Barr nuclear antigens (EBNA1, 2, 3A/3, 3B/4, 3C/6 and -LP), three latent membrane proteins (LMP1, 2A and 2B), two small noncoding RNAs (EBER1 and EBER2) and several dozen microRNAs (11–13). Based on the gene expression profiles, four different viral latency programs - latency 0, I, II, and III are recognized (**Figure 1**). Latency 0 is typically found in memory B-cells and is characterized by the expression of only EBERs. In latency I, EBERs and EBNA1 are expressed. Latency II is characterized by EBERs, EBNA1, LMP1, and LMP2. Latency III shows the broadest expression pattern, adding EBNA2, EBNA3, and EBNA-leader protein (EBNA-LP) to the proteins expressed in latency II (14). Reactivation occurs when EBV enters the replicative cycle marked by the expression of lytic genes, including its own transcription factor and DNA polymerase catalytic subunit, which results in amplification of its genome by more than a 100 fold (15). The process is initiated by transcriptional activation of BZLF1 and BRLF1, the corresponding protein products of which function as transcription activators, thereby triggering the initial lytic stimulus (16).

In addition to the well-established etiological role of EBV in malignancies, accumulating and creditable evidence indicates that this virus is also involved in the pathogenesis of several autoimmune diseases, including multiple sclerosis (MS) (18–20), lupus erythematosus (SLE) (21, 22) and rheumatoid arthritis (RA) (23). A recent landmark longitudinal study provided compelling evidence for a strong association between EBV infection and the subsequent development of MS (18). The study involved testing more than 10 million active US military personnel over a course of 20 years. During this period, 801 individuals developed MS, 35 of which were initially EBV seronegative, but all seroconverted before the onset of MS, except for one case. Thus, EBV infection increased the risk of MS by 32-fold. No such risk was found with CMV or other viruses (18). However, it is important to note that EBV infection alone is insufficient to cause MS, as the vast majority of EBV infected individuals never develop the disease. It appears that EBV acts within a complex network of genetic, environmental, and immunological factors, functioning as a trigger or disease modifier in susceptible individuals (24). Recognizing this distinction is important for interpreting epidemiological associations and developing effective preventive strategies.

Understanding why this common virus triggers some individuals to develop MS, while others to develop SLE, RA, or remain disease-free despite lifelong infection remains unresolved. Thus, a comprehensive and comparative framework examining shared versus disease-specific EBV-driven pathogenic mechanisms is of interest. This review examines the association between EBV and three autoimmune diseases - SLE, RA, and MS, focusing on the underlying molecular mechanisms.

## 2 Shared mechanisms in EBV-driven autoimmunity

### 2.1 EBV latent proteins as common triggers

#### 2.1.1 EBNA1 as a universal autoimmune initiator

Amongst the several lytic and latent EBV gene products, the latency-associated EBNA1 is the only protein consistently expressed in infected B-cells in healthy carriers (25). Interestingly, all three diseases (MS, SLE, RA) demonstrate elevated antibody responses to EBNA1, indicating this protein to be a potential shared trigger of autoimmunity (26). EBNA1 employs multiple mechanisms to prevent presentation of its peptides on the surface of EBV-infected B-cells (27). However, its large glycine–alanine repeat domain promotes T-cell independent antibody responses that facilitate epitope spreading (28). More recently, it was reported that EBNA1-specific CD4^+^ T-cells can cross-react with MS autoantigen ANO2, thereby leading to EBV-mediated neuroinflammation and MS pathology (29). This EBNA1-specific heteroimmune responses appear particularly important because this protein is expressed in all EBV-infected B-cells in latency programs I-III, suggesting that it may be a trigger for immune dysregulation.

#### 2.1.2 LMP1 and LMP2 - CD40 like mimicry and B-cell dysfunction

LMP1, initially noted for its oncogenic activity, is also known for functioning as a CD40 homolog and represents a key functional mimic driving aberrant B-cell activation across autoimmune diseases (30). Unlike CD40, which requires CD154-mediated trimerization for activation, LMP1 self-aggregates through its six transmembrane domains in a ligand-independent manner, driving constitutive downstream signaling, promoting B-cell activation, germinal center formation, and antibody and cytokine production (31). It also upregulates activation-induced cytidine deaminase (AID) which drives polyclonal B-cell activation and class-switching recombination, rescuing autoreactive B-cells from apoptosis and promoting auto-antibody production across multiple diseases (32). By signaling independently of T-cell–derived CD154, LMP1 enables constitutive B-cell activation, a mechanism that may facilitate immune evasion and drive the loss of immune tolerance, leading to autoimmunity. In SLE specifically, LMP1 expression in the context of EBNA1 molecular mimicry amplifies immune dysregulation, promoting enhanced cellular and humoral responses that cross-react with lupus autoantigens (particularly Sm) (30).

Additionally, LMP2A has been reported to enhance the antigen presenting function of B-cells, which increases autoreactive T-cell activation, promotes inflammation and facilitates disease progression in the animal model of experimental autoimmune encephalomyelitis (EAE) (33). LMP2A also induces anti-Sm B-cell responses, enhances B-cell sensitivity to toll like receptor (TLR) stimulation, and influences plasma cell differentiation of otherwise regulated B-cells. By lowering the activation threshold through augmented B-cell receptor (BCR)-TLR signaling, LMP2A facilitates the survival and activation of autoreactive B-cells, ultimately promoting autoantibody production, and contributing to EBV-associated autoimmune diseases (34).

#### 2.1.3 EBNA2: transcriptional activation and viral persistence

EBNA2 is the principal transcriptional activator during EBV latency. Activating the EBV Cp promoter regulates the expression of other EBNAs, as well as the LMP1 promoter (35). It interacts with several transcription factors and binds to a large proportion of genetic loci associated with autoimmune disease risk. Notably, NF-κB subunits such as RELA, RELB, NFKB1, and NFKB2 significantly overlap with these regions, highlighting a potential role for EBNA2-mediated NF-κB signaling in driving susceptibility to inflammatory autoimmune disorders (36).

### 2.2 Molecular mimicry in autoimmunity

Mimicry of host protein structures is a common mechanism employed by viruses to evade the host’s immune system (37). However, for such mimicry to be evolutionarily favorable, the benefits must outweigh the costs, such as longer replication time due to increased protein length, or potential loss of function arising from mutations required to achieve mimicry (mimicry trade-off hypothesis) (38). To combat this, viruses employ short peptide sequences, typically 3 to 10 amino acids long, to mimic host cellular motifs to allow effective immune evasion and limit negative impacts on protein function or length. This is referred to as short linear mimicry, a process that offers an optimal solution to the mimicry trade-off, and benefits viral survival (39, 40). Viruses belonging to the Herpesviridae and Poxviridae families have been shown to exhibit this type of mimicry (37). That said, unlike herpesviruses, poxviruses do not seem to be associated with any autoimmune disease. This is explained by their tendency to mimic a narrower set of host proteins due to its targeting of longer and accurate mimics, while herpesviruses like EBV, appear to target short and low accuracy mimics. In other words, poxviruses mimic precisely but selectively whereas herpesviruses mimic broadly but imperfectly, and that broader mimicry may disrupt immune tolerance to a greater extent. Coupled with this is the ability of herpesviruses to establish latency, which increases the frequency of targeting cross-reactive epitopes, and thereby the likelihood of autoimmune sequelae. It has also been reported that EBV latent proteins display significantly more mimicry than those expressed during the lytic phase. However, this observation is not universal to all herpesviruses; HHV8 and CMV appear to display high levels of mimicry in both, their latent and lytic proteins (37).

Even though molecular mimicry between the various EBV epitopes and host antigens represent a fundamental shared mechanism across several autoimmune disorders, the specific host targets differ dramatically by disease (**Table 1**). This divergence suggests that although the viral mimicry mechanism is central to autoimmunity, it is the tissue-specific distribution of corresponding host proteins that largely dictate the disease phenotype.

**Table 1:** Proteins involved in molecular mimicry in MS, SLE and RA.

Disease	EBV component	Host proteins	References
Multiple Sclerosis (MS)	EBNA1	GlialCAM, myelin basic protein, αB-crystallin, anoctamin-2	(20, 29, 41, 42)
Systemic Lupus Erythematosus (SLE)	EBNA1	SmB, SmD, C1q, Ro, p542, dsDNA	(43–48)
Rheumatoid Arthritis (RA)	EBNA1, EBNA2, vIL-10	Synovial membrane 62-kDa protein, cytokeratin and type II collagen, hIL-10	(49–51)

### 2.3 Autoreactive B-cells and autoimmunity

Early B-cell development includes a population of autoreactive naïve B-cells that are usually kept in check by central and peripheral B-cell tolerance checkpoints. Central B-cell tolerance in humans relies on proper BCR and TLR signaling and function, while the peripheral B-cell tolerance checkpoints depends on T-cells/Tregs to prevent their accumulation and subsequent production of self-reactive antibodies (52).

When EBV infects these autoreactive B-cells, it drives their proliferation and persistence as apoptosis-resistant, latently infected memory B-cells in genetically susceptible individuals. These cells accumulate in target organs, and act as antigen-presenting cells (53). Cross-reactive CD4^+^ T-cells activated in lymphoid tissues migrate to these sites, receive survival signals, and evade apoptosis, leading to sustained inflammation, cytokine production, and chronic tissue damage, as seen in autoimmune diseases (54). The subsequent antibody production in the host has been shown to exhibit three important features: IgM dominance facilitates target cell injury via activation of the classical complement pathway; ubiquitous immunoglobulin production independent of germinal centers or bone marrow; and rescue of autoreactive B-cells from checkpoints (55). All three processes skew the repertoire toward autoantibody production. Thus, EBV reprograms the autoreactive B-cells into activated antigen presenting cells. These cells in turn stimulate autoreactive helper T-cells, further activating more autoreactive B-cells, including uninfected ones. In this scenario, the B-cells effectively act like triggers that sets off an inflammatory cascade and orchestrate the activation of a systemic autoimmune response (56).

### 2.4 Type 1 interferon dysregulation

Type 1 interferon (IFN-1) activation represents a pathogenic mechanism across MS, SLE, and RA. EBV nucleic acids and viral proteins trigger pattern recognition receptors (TLRs, RIG-I-like receptors, cGAS-STING pathway) leading to a dysregulated IFN-1 release (57). In fact, some TLRs are more specifically associated with some autoimmune diseases. For example, TLR2 or TLR4 polymorphisms are frequently associated with RA, while TLR5 and TLR8 polymorphisms are associated with SLE and Sjogren’s syndrome (58). IFN-targeting therapy in SLE has in fact shown positive results in phase II and III clinical trials, underscoring its pathogenic role in autoimmunity (59–61).

## 3 Disease-specific mechanisms: selective vulnerabilities

Although MS, SLE, and RA share several EBV-driven pathogenic mechanisms that trigger an autoimmune response, disease-specific outcomes reflect the interplay between several viral, environmental, and host factors. Differences in the localization of persistent EBV infection, as seen in a compartmentalized immune response in CNS in MS, a higher systemic viral load in SLE and persistence within synovium in RA, may influence the tissues targeted by immune responses. Similarly, the timing of primary infection and immune status during viral reactivation can shape the magnitude of antiviral response. For example, delayed EBV infection to adolescence and the development of infectious mononucleosis (IM) has been consistently shown to be associated with 2-3-fold increase risk in MS (62, 63). Similar associations may also exist for SLE and RA, but the data is far less clear. Reports indicate that SLE is more frequent and more severe in immigrants of African and Asian origin living in Europe and North America (64, 65). Whether is this is due to delayed infection or underlying genetic susceptibility or other environmental factors is unclear (66–68). Finally, host genetic factors, particularly HLA-associated antigen presentation, help determine the specificity of autoreactive responses. Together, these factors provide a background for understanding how EBV-driven immune response can give rise to distinct autoimmune phenotypes. This complexity reinforces the need for a disease-specific analysis to achieve a more integrated view of EBV-associated autoimmune disease.

### 3.1 Multiple sclerosis

Multiple sclerosis is a chronic autoimmune disease of the central nervous system (CNS) characterized by mononuclear cell infiltration, demyelination and neurological dysfunction (69). The environmental risk factors most consistently linked to MS are infection with EBV, sun exposure/vitamin D deficiency, and smoking (70–72). Although the exact mechanism of how EBV induces or drives MS has not been fully delineated, several aspects are now becoming clearer.

#### 3.1.1 Role of CNS-tropic CD8^+^ T-cells

CD8^+^ T-cells play a central role in controlling EBV infection by eliminating infected B-cells directly through cytotoxic mechanisms (73). CD8^+^ T-cells specific for the EBNA1-derived epitope HPVGEADYFEY were detected in both the CSF and blood of patients with MS and Other Inflammatory Neurological Diseases (OIND) using HLA-matched pentamer staining (74). The numbers of CD8^+^ T-cells were particularly elevated in the CSF compared to peripheral blood, irrespective of the diagnosis. This suggests that there is a preferential access of EBV specific CD8^+^ T-cells to the brain compared to other common viruses such as CMV. However, the frequency of EBV-specific CD8^+^ T-cells did not significantly differ between MS and OIND (74). A similar heightened CD8^+^ T-cell activation against EBV, but not CMV was observed, supporting a role for EBV in the onset of MS. Interestingly, no such difference were found between patients with MS and OIND in terms of EBV-specific CD4^+^ T-cell response (75).

In addition to the trafficking of CD8^+^ T lymphocytes, there is evidence of clonal proliferation of these cells within the CNS in MS patients. High throughput sequencing of TCR-β repertoires revealed that the identity of T-cell clones that are expanded in CSF was different from those in blood in MS patients, when compared to those in OIND (76). While EBV drives the trafficking of T lymphocytes to the brain, clonal proliferation may not necessarily require the continual presence of the viral antigen. Studies show that clonal expansion could also be secondary to a continual recognition of neuron-specific neoantigens sourced from damaged or stressed neural cells. The neural cells which normally do not constitutively express MHC class I molecules, present autoantigens secondary to injury during demyelination. They are then recognized by infiltrating autoreactive cytotoxic T-lymphocytes, which accumulate within and around CNS lesions (77). Additionally, the number of CD8^+^ T-cells within MS lesions also seem to correlate with active axonal injury, which in turn correlates with disease disability (78).

Further characterization of T-cell clonotypes using single-cell RNA-sequencing and T-cell receptor-sequencing analysis of the CSF and blood from individuals with MS and control cohorts, demonstrated that these T-cells are highly differentiated, antigen-experienced with cytotoxic phenotype and possess high CNS-trafficking potential. Gene expression profiling shows dramatic differences in the cells from CSF versus peripheral blood. The former show upregulation of genes associated with migration and trafficking (CXCR3, CXCR4, CCL4, ITGB1 and ITGA4) as well as cytotoxicity (GZMK and GZMA), while the peripheral blood cells display an alternate activation state with higher levels of FOS, JUN, DUSP1 and GADD45B (79). This suggests that CNS harbors an activated clone of CD8^+^ T-cells specific for EBV in MS patients.

While substantial evidence supports a cytotoxic role for CD8^+^ T-cells in MS, a dysfunctional phenotype has also been reported (80). When the behavior of effector CD8^+^ T lymphocytes (CD8^+^ CD57^+^ T-cells) in healthy donors and MS patients during EBV infection was studied, it was demonstrated that these cytotoxic T-cells eliminated the virus in healthy donors and those with active disease but failed to do so in MS patients with inactive disease (80). Instead, an anergic exhaustion-like phenotype with high levels of PD-1 expressing T lymphocytes was observed in the latter, exhibiting weak response to TCR stimulation and impeded release of perforin and granzyme B. This suggests that it is during remission that EBV reactivation occurs, owing to inefficient viral control and subsequent worsening of the disease. During active disease, the CD8^+^ CD57^+^ T-cells maybe be able to clear the virus to an extent, but it is the repeated antigenic stimulation and inflammatory milieu that drives the upregulation of PD1 and switch to anergy phenotype, rendering it incapable of cytotoxicity (80). The tendency of EBV for prolonged latency programs and immune evasion probably allows sufficient time for the host to suffer from impaired surveillance, which runs parallel to the long course of the disease itself.

#### 3.1.2 Role of EBNA1-CNS protein mimicry

**Figure 2:**
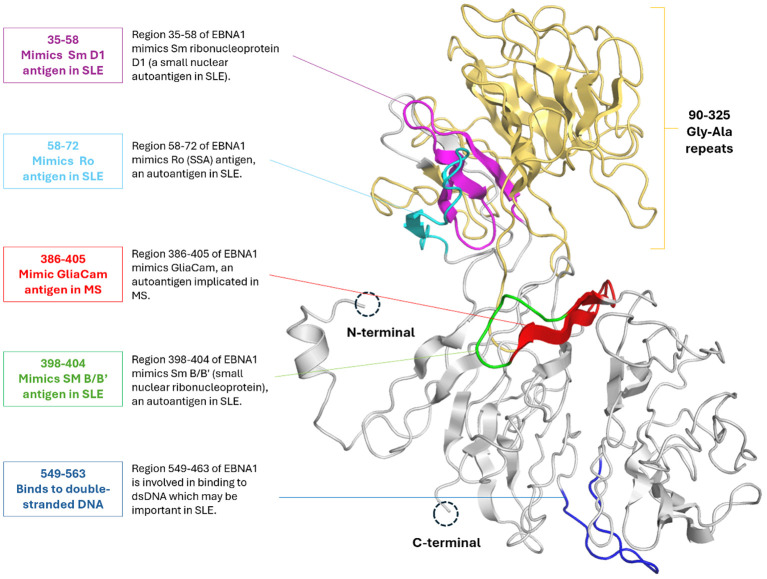
Predicted EBNA1 structure, highlighting some of its epitopes that mimic autoantigens. The complete EBNA1 protein has not been crystalized. Hence, its full-length 3D structure is not available. To predict its structure, we downloaded the full-length EBNA1 amino acid sequence from UniProt and used it as the query sequence in the I-TASSER web-server (85). Further structural visualization and figure preparation were performed using PyMOL (https://www.pymol.org/) to highlight the relevant regions involved in autoimmunity.

EBNA1 contains an immunogenic region (380-450aa) located between the second glycine–arginine repeats and the C-terminal DNA-binding domain (81). Studies have shown that antibodies against this region of EBNA1, cross-reacts with multiple CNS proteins, including myelin basic protein (MBP), anoctamin-2 (ANO2), αB-crystallin (CRYAB) and glial cell adhesion molecule (GlialCAM) (41, 82, 83) (**Figure 2**). In fact, the presence of elevated EBNA1-specific antibody responses against three or more cross-reactive peptides, increased the risk for MS by 1,366-fold (83). Indeed, anti-EBNA1 antibodies beyond 380-450aa region can also cross react with cellular proteins, consistent with intramolecular epitope spreading and an expanded antibody response (84). In another study, a monoclonal antibody was isolated from the CSF of a patient with MS which bound to the MS-associated EBNA1 (386–405) epitope with high affinity and cross-reacted with GlialCAM. Moreover, immunization of EAE mice with this EBNA1 (386-405) peptide exacerbated autoimmune demyelination, providing a mechanistic link between EBV and development of demyelination (20). The inefficient viral control due to dysfunctional CD8^+^ T lymphocytes prolongs the presentation of viral antigens, allowing sufficient time for immune tolerance to break down, driving the production of autoantibodies.

#### 3.1.3 Cross-reactive CD4^+^ T-cell epitopes

Although antibody-mediated molecular mimicry between EBNA1 and various CNS antigens provides evidence for EBV-driven autoreactivity, recent studies suggest that cross-reactive CD4^+^ T-cell responses are also of pathogenic significance. Unlike antibodies, autoreactive CD4^+^ T-cells being directly linked to the major MS susceptibility allele HLA-DRB1*15:01, are capable of initiating CNS inflammation while simultaneously providing help to autoreactive B-cells (34). It is also plausible that EBV-induced CD4^+^ T-cell cross-reactivity acts as an initiating event due to genetic susceptibility, and B-cell derived cross-reactive antibodies contribute to amplification and perpetuation of disease. More recently, cross-reactive T-cell responses have also been identified between EBNA1 and CRYAB and ANO2, in addition to antibody mediated mimicry (29, 82).

#### 3.1.4 Role of meningeal tertiary lymphoid structures (TLSs)

The study by Magliozzi et al. (86) was one of the first to show an association between ectopic lymphoid tissue formation in the CNS and the clinical course and extent of tissue destruction in MS (86). By analyzing post-mortem brain tissues from MS cases, they showed that in secondary progressive MS (SPMS), inflamed meninges contained structures resembling secondary B-cell follicles with germinal centers composed of proliferating B-cells, plasma cells, T-cells, and follicular dendritic cells (FDCs). These aggregates were capable of activating the molecular machinery to sustain *in situ* antibody diversification, isotype switching, B-cell differentiation, and oligoclonal expansion. This enabled the formation of active ectopic germinal centers, which could also support the production of autoreactive plasma cells at the local site of inflammation (86). These findings indicated that the inflamed CNS can sustain local B-cell responses through the formation of ectopic lymphoid tissue as seen in other autoimmune diseases, such as Hashimoto’s thyroiditis (87), myasthenia gravis (88), and Sjögren’s syndrome (89). These ectopic germinal centers produce disease-relevant antibodies, thereby amplifying the local autoimmune responses.

In the literature, the term ectopic lymphoid tissue has been used interchangeably with ectopic lymphoid structures and tertiary lymphoid structures (ELS, TLS). In contrast to TLS in other tissues like the salivary glands of Sjogren’s disease patients or the joints of rheumatoid arthritis patients, T-cell and B-cell zones in the leptomeningeal TLS are less defined with lack of evidence for bona fide germinal center reactions (90). Nevertheless, leptomeningeal TLS are seen to be spatially associated with a peculiar grey matter lesion called Type III lesion, unique to MS (91). These lesions localize towards the cortex, forming ribbon-like patterns across multiple gyri, and represent axonal, neuronal and synaptic injury/loss, in addition to demyelination (90). The chemokine CXCL13, known for the recruitment and retention of CXCR5^+^ lymphocytes, as well as both B-cells and T follicular helper (Tfh) cells are also demonstrated within these structures (92). Furthermore, RORγt^+^ Th17 cells, capable of producing IL-17, were found to be preferentially enriched within the TLS, rather than a diffuse meningeal infiltration, underscoring its role in the organized development of TLS (93). This CNS-compartmentalized pathology distinguishes MS from SLE and RA, where EBV dysregulation is predominantly systemic.

Binding of EBNA2 to MS risk loci: EBNA2 regulates MS risk gene transcription in EBV infected B-cells through an allelic imbalance in binding at 5 out of 6 MS risk loci - namely TRAF3 (*p* < 0·05), CD40 (*p* < 0·001), CLECL1 (*p* < 0·0001), TNFAIP8 (*p* < 0·001) and TNFRSF1A (*p* < 0·001) (94). Many of these genes modulate B-cell activation and survival in response to EBV infection, suggesting that host’s ability to control the viral burden does play an important role in determining susceptibility to MS.

#### 3.1.5 EBV-HERV relationship

Human endogenous retroviruses (HERVs) are remnants of ancient retroviral integrations within the human genome that can be transcriptionally reactivated by various cellular stimuli. Emerging evidence suggests a functional link between EBV and HERV activation, particularly involving ERVW-1, which encodes the envelope protein Syncytin-1. Low-level ERVW-1 transcription is increased during EBV lytic reactivation, and Syncytin-1 appears to act as a pro-lytic factor, suggesting that it contributes to lytic reactivation of EBV and maintaining persistently high viral load in the host (95). This relationship was also observed in several other studies, underscoring the complex interactions of EBV, HERV and host immunity (96–98).

Despite multiple mechanistic studies delineating a role for EBV in MS pathogenesis, some reports have yielded inconsistent findings. In an early case controlled study that used global gene expression profiling to identify potential genes involved in the pathogenesis of MS, reported that although there is striking upregulation of Ig related genes in the cortex of the brain in MS patients, there was no detection of EBV by qPCR or immunohistochemistry (99). Similarly, only 1 amongst 48 MS patients showed EBV positivity in their CSF (100). By contrast, a more recent large study involving over a thousand brain samples from MS and non-MS cases, reported the detection of EBV in 90% of the MS cases (101). Moreover, the virus was directly localized to the brain and shown to be transcriptionally active. Although the evidence linking EBV to the pathology of MS is now substantial and very credible, the exact mechanism by which this common virus triggers and drives MS remains to be elucidated (102). In this context, the recent development of a rabbit model of EBV infection may prove to be a great tool in addressing some of these mechanistic questions in a controlled manner (103, 104). Using this model, it has been shown that introduction of the virus via intravenous injection, results in the virus crossing the blood-brain barrier and inducing massive inflammatory follicle-like structures consisting of macrophages and microglia surrounded by lymphocytes and astrocytes (105). Importantly, these inflammatory aggregates, which can be as large as 500-600µm, show well-defined demyelination. However, a definite proof that EBV is causing MS, may come from vaccination against the virus (106). Several vaccines against EBV are currently in development, including an mRNA-based vaccine (107–109).

#### 3.1.6 Therapeutic implications

The identification of EBNA1–GlialCAM cross-reactivity and HLA-DR15-restricted autoreactive T-cell repertoires provides a rationale for antigen-specific therapeutic approaches in MS, including peptide tolerization, tolerogenic dendritic cells, and EBNA1-directed immunotherapies (20, 110). EBNA1-targeting vaccines have also emerged as a potential preventive strategy, particularly given the evidence supporting EBV as a necessary antecedent of MS (18, 102). EBV infected B-cells have been shown to capture and present myelin antigens, providing a mechanistic link between viral persistence and autoreactive T-cell activation within the CNS. This model is consistent with the remarkable efficacy of B-cell-depleting therapies, such as anti-CD20 monoclonal antibodies, which may exert part of their therapeutic effect through the elimination of EBV-infected memory B-cells (19, 111, 112).

### 3.2 Systemic lupus erythematosus

Studies have shown that patients with SLE exhibit approximately a 40-fold higher EBV viral load in peripheral blood compared with healthy controls, independent of B-cell counts, medication use, disease activity, or lupus nephritis status, underscoring the virus’ role in SLE pathogenesis (113). The lifelong, intermittently reactivating pattern of EBV infection provides repeated immune exposure, adding to which is EBV’s tropism for B-cells - the key producers of autoantibodies. This creates a very permissive environment for B-cell activation, survival, and dysregulated antibody production (114). Although the details of the role of EBV in the pathogenesis of SLE is poorly understood, the current evidence paints a complex picture.

#### 3.2.1 Role of lupus-specific autoantigen mimicry

Several lupus-associated autoantigens and corresponding EBV EBNA1 epitopes have been described in the literature, with their homology driving autoantibody production in SLE as shown in **Table 2**, with epitopes mapped in **Figure 2**.

**Table 2:** Molecular mimicry between major lupus associated autoantigens and EBV EBNA1 epitopes.

Host nuclear antigen	Host antigen amino acid sequence	EBNA1 epitope (amino acid position)	EBNA1 epitope amino acid sequence	References
Sm B/B’	PPPGMRPP	EBNA1 (398–404)	PPPGRRP	(30, 45)
Sm D1	AGRGRGRGRGRGRGRGRGRGGPRR	EBNA1 (35-58)	GGDNHGRGRGRGRGRGGGRPGAPG	(46)
Ro	TKYKQRNGWSHK	EBNA1 (58-72)	GGSGSGPRHRDGVRR	(47)
C1q	GRPGRRGRPGLKG	EBNA1 (348-364)	GSGGRRGRGRERARGRS	(43)
p542	GGGASGGGGGGGSGGGGSGGGGGGSS	EBNA1(90-325)	Gly-Ala repeats	(44, 115, 116)
dsDNA	*	EBNA1(549− 563)	PQPGPLRESIVCYFM	(48, 117)

*mimicry between EBNA1 and dsDNA is mediated by structural similarities rather than sequence homology.

- Anti-Sm antibody: Sm proteins (Sm B/B′, D1, D2, and D3) are core spliceosomal components which also turn out to be major autoantigens in SLE (114). A dominant immunogenic epitope within Sm B/B′ - PPPGMRPP motif, shares homology with EBNA1 – PPPGRRP. This EBNA1 epitope is preferentially recognized by lupus sera, and immunization with either the Sm or EBNA1 peptide induces lupus-like autoimmunity (30, 45). Similarly, Sm D1 (95-119aa) exhibits sequence homology with EBNA1 epitope 35-58aa; antibodies from lupus patients cross-react with both peptides, and immunization with EBNA1 (35-58aa) elicits antibodies recognizing Sm D1 (46).
- Anti-Ro antibody: Molecular mimicry has also been reported between EBNA1 and a 60kDa RNA-binding autoantigen Ro (47). The immunodominant Ro epitope (169-180aa) cross-reacts with EBNA1 epitope 58-72aa, and immunization with either peptide induces lupus-associated autoimmunity and clinical manifestations. Notably, the autoantibody response appears to occur even before the onset of clinical disease, suggesting that EBV could be an important player in the initiation of SLE (47).
- Anti-C1q antibody: Loss of functional mutations in C1q, the starter molecule of the complement classical pathway is a rare but strong susceptibility factor for the development of SLE (118). Antibodies specific for the EBNA1-derived peptide (348-364aa) can cross-react with region of C1q, a yet another demonstration of molecular mimicry at play in the pathogenesis of SLE (43).
- Anti-p542 antibody: The autoantigen p542, involved in pre-mRNA splicing and mRNA metabolism contains a glycine-rich 28-mer homologous to glycine/alanine repeat peptide in EBNA1 (90-325aa), further substantiating the role for molecular mimicry in SLE pathogenesis (44, 115, 116).
- Anti-dsDNA and anti-nuclear antibody (ANA): Double-stranded DNA (dsDNA) constitutes another class of autoantigens for which EBV-derived mimotopes have been documented (119). Experimental data on mice showed that the expression of full-length EBNA1 via a vector, induced anti-dsDNA antibodies (120). In an attempt to map this specific epitope within EBNA1, a 15-amino acid peptide called PFM-15 was identified within the carboxyl-terminal region of EBNA1, the antibodies against which cross-react with dsDNA (48, 117). Furthermore, a notable increase in the levels of antibodies against viral capsid antigen (VCA) and early antigen (EA) were found in newly transitioned relatives of SLE (ANA+) compared with non-transitioned relatives (ANA-), suggesting that heightened serologic reactivation of EBV increases the probability of transitioning to SLE in unaffected SLE relatives (121).

#### 3.2.2 EBNA2 mediated epigenetic and transcriptional remodeling

EBNA2 binds to regulatory regions of CD27, CD70, ZEB2, and TBX21 and promotes the development of CD27^+^ CD21^low^ ZEB2^+^ T-bet^+^ activated memory B-cells. Together with the upregulation of APC-related genes in EBV^+^ B-cells, EBV remodels autoreactive ANA^+^ B-cells into hyper efficient antigen-presenting cells that continually activate T helper cells and differentiate into antibody-secreting plasma cells (56).

#### 3.2.3 Role of systemic multi-organ involvement

Recent studies show that recurrent EBV reactivation amplifies systemic inflammation in SLE. This is brought about by EBV-driven activation of plasmacytoid dendritic cells which promote production of pro-inflammatory type I interferon (122). At the same time, EBV IL-10 impairs monocyte-mediated apoptotic cell clearance, facilitating immune complex formation and further stimulating IFNα release. This exaggerated IFN release, and impaired apoptotic clearance, drives a systemic inflammatory response, causing cumulative damage to tissues and organs, such as the skin, joints, and kidneys (122).

It is still debated whether EBV reactivation is a cause or consequence of SLE disease activity. In a study of adults with established disease, frequent EBV reactivation appeared to be an aggravating consequence, rather than a cause of SLE. EBV-specific CD8^+^ T-cell responses in SLE patients were found to have marked impairment in the exocytosis of cytotoxic granules secondary to upregulation of PD-1 as observed in the brain in MS (123). Thus, it is plausible that autoimmune-driven B-cell activation in SLE triggers EBV lytic reactivation, leading to episodic viral replication and expansion of EBV-specific T-cells. These T-cells cross-react with self-antigens and, together with EBV-induced type I IFN responses, amplify autoimmunity. Repeated reactivation may induce PD-1–mediated T-cell exhaustion, limiting tissue damage but impairing viral control and allowing persistent EBV replication.

#### 3.2.4 Therapeutic implications

In SLE, persistent type I interferon activation and EBV-mediated reprogramming of autoreactive B-cells into APC-like pathogenic cells provide a strong rationale for therapies targeting IFN and B-cell pathways (56, 124). The clinical efficacy of the anti-IFNAR1 monoclonal antibody anifrolumab further supports the pathogenic role of IFN-I dysregulation in lupus (125). Similarly, baricitinib and other JAK inhibitors may attenuate downstream IFN signaling and inflammatory amplification pathways (61). B-cell-directed therapies, including anti-CD20 and anti-BAFF agents, may also counteract EBV-associated autoreactive B-cell survival and differentiation (126, 127).

### 3.3 Rheumatoid arthritis

Like MS, some of the early studies linking EBV to RA were also based on seroepidemiologic observations (128, 129). Patients with RA had relatively high levels of antibodies that reacted to EBV proteins, particularly EBNA1. It was also reported that RA patients had defective T-cell regulation and hence higher proliferating EBV infected lymphocytes and viral load (130). More recent studies attempted to understand the molecular and cellular mechanisms underlying some of these early observations.

#### 3.3.1 Role of ectopic lymphoid structures

ELS appear to be a common feature in the synovium of RA patients (131, 132). These ELS are not uniform, but rather a spectrum, range from diffuse immune infiltration to semi-organized cellular aggregates, to well-organized structures containing germinal center like features. However, the details of how these ELS are triggered and what their role is in the pathogenesis of RA remains poorly understood. In an attempt to understand the link between EBV and ELS, Croia et al. (133), examined 43 synovial tissues from RA patients with and without ELS. The findings revealed that EBV was present exclusively in ELS-positive synovium, as demonstrated by the expression of EBV latent proteins, presence of EBER+ cells, and immunoreactivity for EBV latent and lytic antigens. Notably, this finding was absent in the 11 osteoarthritis control synovia and in RA synovia with diffuse inflammatory infiltrates. This preferential homing of EBV infected B-cells to these specialized lymphoid structures could induce the expansion and differentiation of autoreactive B-cells into autoantibody-producing plasma cells (133). This behavior of EBV is also observed in MS and myasthenia gravis patients, where viral persistence is associated with lymphoid neogenesis, suggesting that EBV colonization of ELS could be a signature of organ-specific autoimmunity (134, 135).

#### 3.3.2 Role of antigen mimicry and cytokine dysregulation

EBV antigens undergo post-translation citrullination, thus becoming targets for a highly specific RA biomarker, anti-citrullinated protein antibody (ACPA) which cross-reacts with citrullinated fibrinogen in inflamed synovial tissue (136). Antibodies against EBNA1 have been shown cross-react with proteoglycan peptides essential for cartilage integrity, including cytokeratin, type II collagen, and a 62-kDa protein of the synovial lining (49, 137). The net effect of this is inflammation and joint injury. In addition to EBNA1, Fanelli et al. (138) observed that EBNA2, particularly EBNA2-A, exhibited strong reactivity with ~70% of RA sera, with minimal reactivity to EBNA1 and EBNA3 peptides, indicating that citrullinated EBNA2 could serve as a promising ACPA target (138). Molecular mimicry is also not restricted to the EBNAs. The EBV-encoded interleukin-10 (vIL-10) produced during the lytic phase of the virus exhibits 83% sequence resemblance with human interleukin-10 (hIL-10), which is vital in promoting the differentiation of regulatory T-cells (139, 140). Antibodies to hIL-10 consequently breaks the immune tolerance, initiating an exacerbated, self-targeting humoral response (51, 141).

EBV primes B- and T-cells to cross-react with synovial antigens which in genetically susceptible individuals, promote activation of autoreactive lymphocytes, including B-cells to produce rheumatoid factor, ACPA, and joint specific antibodies. This in turn triggers the release of a storm of inflammatory cytokines like TNF-α, IL-1β, IL-6, IL-17. While mimicry may initiate the response, persistent inflammation drives epitope spreading and amplifies autoimmunity (142). The level of the antibodies against these Viral Citrullinated Peptides (anti-VCP antibodies) also correlate with other RA-related markers like anti–cyclic citrullinated peptide antibodies, rheumatoid factor and erythrocyte sedimentation rate, supporting their utility in distinguishing RA from other chronic arthritis and connective tissue disorders (51).

#### 3.3.3 Role of ACPA-associated autoimmunity

A large proportion of ACPA-producing plasma cells at the periphery of ectopic germinal centers appear to be infected with EBV (133). These findings raise the possibility that EBV infection may favor the survival of pathogenic autoreactive B-cells that participate in the formation of ectopic germinal centers in the RA synovium. ELS and the local availability of self-antigens likely provide the appropriate environment that allows EBV-infected autoreactive B-cells to selectively expand, undergo affinity maturation and differentiate into autoantibody-producing plasma cells that home to the RA synovium (143).

#### 3.3.4 Therapeutic implications

Given that ACPA responses frequently precede the onset of clinical arthritis by several years, the recognition of citrullinated EBV epitopes by RA sera raises the possibility that EBV contributes to the early breach of tolerance and expansion of autoreactive B-cell responses during the preclinical phase of disease (138, 144, 145). Although direct interventional evidence is currently lacking, future studies should explore whether EBV monitoring, vaccination, or antiviral approaches could complement existing strategies, particularly during the preclinical stages of disease.

**Figure 3:**
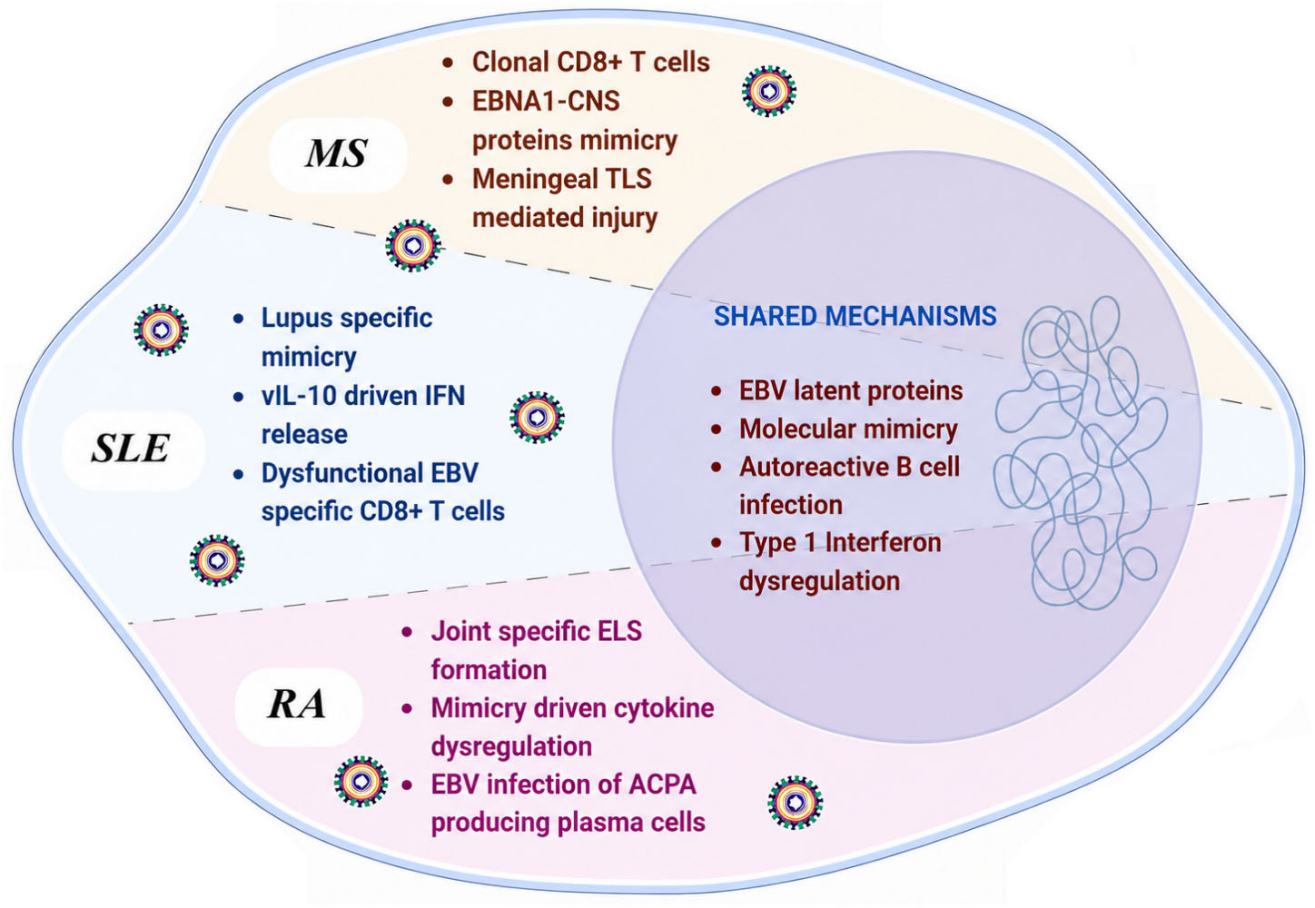
Shared and disease-specific mechanisms of EBV-driven autoimmunity. EBV establishes latent infection in memory B-cells and promotes autoimmunity through shared and disease-specific pathways. The central overlapping region (purple) represents shared mechanisms, including involvement of EBV latent proteins, molecular mimicry, autoreactive B-cell infection, and type 1 interferon dysregulation. Disease-specific features in MS (beige) are characterized by CNS-targeted CD8^+^ T-cells, EBNA1-CNS mimicry, and meningeal TLS. In SLE (blue), specific features include lupus specific mimicry, vIL-10 driven IFN responses, and dysfunctional EBV-specific CD8^+^ T-cells. In RA (pink), features include presence of synovial ELS, cytokine dysregulation, and EBV-infected ACPA-producing plasma cells. These findings illustrate how EBV-driven pathways give rise to distinct autoimmune phenotypes.

Significant progress has been made in showing that EBV is an etiologically risk factor in the pathogenesis of autoimmune diseases such as MS, SLE and RA, yet the exact molecular sequence of the mechanisms involved remain to be resolved. The current evidence indicates that these mechanisms are likely to be complex and involve both shared and disease-specific pathways (**Figure 3**).

## 4 Role of HLA in EBV mediated autoimmunity

Despite the interplay of environmental triggers and proposed molecular mechanisms, it is ultimately genetic predisposition, particularly within the HLA locus, which governs susceptibility to disease. HLA does not merely influence the risk, it actively interacts with EBV in influencing the outcome of the disease. A direct molecular link between EBV infection and host genetic susceptibility was demonstrated by enrichment of EBNA2 occupancy at GWAS loci associated with MS, SLE, RA, inflammatory bowel disease, type 1 diabetes, and other autoimmune diseases. This binding of EBNA2 to several autoimmune risk loci show that persistent EBV infection influence disease risk by directly interacting with host genetics that govern immune regulation and antiviral responses.

### 4.1 HLA in MS

HLA-DRB1 gene is highly polymorphic. Genome-wide association studies have identified DRB1*1501, DRB1*03:01, and a group of DRB1*13:03 and DRB1*08:01 haplotypes to be strong risk factors for MS (146). Absence of the protective HLA class I allele HLA-A*02:01 is an additional determinant that elevates MS risk by approximately twofold (84). Studies have shown that patients with HLAB*07+ and DRB1*15+, in the absence of A*02 (HLA-B*07+/DRB1*15+/A*02) demonstrated the highest EBV viral loads. Conversely, the lowest EBV viral loads were seen in MS patients with the opposite HLA profile – HLA B*07−/DRB1*15−/A*02 (147). It is to be noted that HLAB*07 belongs to the same HLA haplotype carrying the HLA-DRB1*15 allele and is believed to only weakly present viral epitopes to cytotoxic T-lymphocytes, thus weakening the immune response and worsening viremia (148). Beyond antigen presentation, HLA-DR also facilitates EBV entry into B-cells, substantiated by binding of the viral protein to its polymorphic region, suggesting that HLA-DR allelic variation directly influences infection outcomes (149). In fact, the combination of EBV as an environmental trigger along with elevated EBNA1 antibodies in HLA DRB1_*_1501 individuals, results in a 24-fold increased risk for MS (150).

Additionally, Wang et al. (110) identified CD4^+^ T-cell clones to be cross-reactive to EBV and autoantigens presented by HLA-DR2a and DR2b within the HLA-DR15 haplotype, indicating that these allomorphs shape an autoreactive T-cell repertoire in MS (110) This, despite a more vigorous CD8^+^ expansion, weakens the immune response to EBV due to ineffective CD4^+^ T-cell clones, resulting in sustained antigenic exposure (151).

### 4.2 HLA in SLE

The HLA locus on chromosome 6 is known for its associations to the occurrence of several autoantibodies in SLE. Antiphospholipid antibodies of SLE are known to be associated with the HLA-DRB1*04/*13 genotypes (152). Characteristic associations between HLA-DRB1*03 allele and autoantibodies to SSA/Ro52/Ro60-SSB/LA antigens, and between HLA-DRB1*15 allele and autoantibodies to nucleosome/SmRNP/dsDNA/RNPA have also been observed (153). HLA-DQB1b is yet another example of allele-dependent binding of EBNA2 to autoimmune-associated genetic variants. It has been shown that EBNA2 and its associated human transcription factors occupy a significant fraction of autoimmune risk loci, thus underscoring the role of EBV antigens in altering the disease risk (36).

### 4.3 HLA in RA

One of the earliest studies to establish a link between EBV and RA susceptibility genes was by Roudier et al. (154). Analysis of EBV protein and DNA databases showed that the EBV glycoprotein 110 (gp110), which is encoded by the BALF4 open reading frame, contains the sequence QKRAAQRAA, which has homology with the RA susceptibility sequence QKRAA located in HLA-DRB1*0401 (154). Several sequence homologies have also been demonstrated between EBNA6 and HLA DQB1*0302 (23). It is also of interest to note that, unlike in MS and SLE, the molecular mimicry in RA occurs between EBV antigens and HLA.

## 5 Conclusion

The role of EBV in autoimmune diseases is complex and intimately intertwined with the immune system at several points. The outcome ultimately depends on tipping the balance between self and non-self. The interplay of the molecular mechanisms by which EBV interacts with the immune system, under the influence of genetic susceptibility, is crucial in determining the disease outcome.

Although there is general acceptance that EBV is involved in the pathogenesis of several autoimmune diseases, the extent of its ability to initiate and drive the disease to full-fledged manifestation remains to be fully elucidated. Addressing this gap could facilitate the development of targeted therapies for early-stage disease and potentially, even prevent it in genetically susceptible individuals. The exact causal relevance of EBV and how it drives demyelination in MS is particularly of interest and requires further investigation. Current evidence points to antigenic mimicry between EBNA1 and cellular proteins to be a central theme in many of these autoimmune diseases. The role of viral strain variation in the pathogenesis of autoimmune diseases is also poorly understood, even though it is relatively well characterized in the context of EBV-associated malignancies (155). Furthermore, larger longitudinal studies are needed to clarify whether EBV reactivation is a cause or consequence of autoimmunity, especially in diseases like SLE. The role of EBV-infected B-cell subsets as drivers of autoreactivity, and their contribution to ectopic lymphoid structures, also warrants deeper investigation.

An additional practical constraint is the dependence on animal models, whose biological differences limit their translational relevance to human EBV infection. Mice do not naturally support EBV infection, thus compelling the use of murine gamma herpes virus, or a humanized mice model (156). Non-human primates, such as rhesus monkeys and Japanese macaques, are infrequently used in research due to limitations related to cost, ethical restrictions and the fact that they are infected with EBV homologs rather than EBV itself. Rabbits on the other hand, can be experimentally infected with EBV, but their B-cell development is grossly distinct, with a shift to peripheral lymphoid tissue in the form of gut-associated lymphoid tissue (GALT) as they age (157, 158). The lack of an ideal animal model poses a challenge for accurately extrapolating these findings to human disease.

From a pharmaceutical standpoint, both preventive (glycoprotein-based vaccines) and therapeutic (T-cell inducing multi-epitope vaccines) strategies targeting EBV are currently under development (159). However, several challenges in the form of complex viral latent and lytic gene expression and variable host genetic profiles stand in the way of its integration into clinical practice. In this era of precision medicine, identifying targeted therapies for these autoimmune disorders remains challenging, yet imperative, particularly given the pervasive and multifaceted involvement of EBV across different pathologies.
